# Successful management of recurrent gestational thrombocytopenia with Chinese herbal medicine: a case report and literature review

**DOI:** 10.3389/fmed.2026.1766226

**Published:** 2026-04-13

**Authors:** Lei Zhang, Qingquan Liu

**Affiliations:** 1 Department of Emergency, Yanqing Hospital of Beijing Chinese Medicine Hospital, Beijing, China; 2 Capital Medical University, Beijing, China; 3 Beijing Hospital of Traditional Chinese Medicine, Capital Medical University, Beijing, China

**Keywords:** case report, Chinese herbal medicine, gestational thrombocytopenia, platelet count, pregnancy

## Abstract

**Background:**

Gestational thrombocytopenia (GT) represents the most prevalent cause of thrombocytopenia during pregnancy, generally manifesting in the third trimester and resolving spontaneously following delivery. Nevertheless, its management primarily involves supportive care, as effective therapeutic options are limited.

**Case presentation:**

A 30-year-old woman experienced recurrent GT in two consecutive pregnancies. Thrombocytopenia developed unusually early, during the first and second trimesters, and progressively worsened. After non-pharmacologic interventions failed to prevent the decline, a modified traditional Chinese herbal decoction was administered. In the second pregnancy, the platelet count increased from 72,000/μL to 135,000/μL after 13 doses, while in the third pregnancy, it rose from 64,000/μL to 111,000/μL after 35 doses. Both pregnancies culminated in full-term vaginal deliveries of healthy infants. The patient experienced no maternal or neonatal complications, and she reported reduced anxiety regarding thrombocytopenia and delivery during follow-up.

**Conclusion:**

This single case suggests that adjunctive traditional Chinese medicine (TCM) was associated with improved platelet counts and enhanced self-reported emotional wellbeing in a woman with recurrent GT, and may inform shared decision-making about mode of delivery. Additional clinical studies are required to confirm the safety and efficacy of TCM for pregnancy-related thrombocytopenia.

## Introduction

Thrombocytopenia, characterized by a platelet count below 150,000/μL, is a prevalent hematologic abnormality during pregnancy, affecting approximately 7–11% of all pregnancies (1). Among these cases, gestational thrombocytopenia (GT) constitutes 70–75% of instances (2, 3). Annually, more than ten million pregnancies worldwide are impacted by GT (4). GT is classified as a diagnosis of exclusion and can only be confirmed when platelet count normalizes following delivery (5). Despite its widespread occurrence, the exact pathophysiological mechanism underlying GT remains inadequately understood, and none of the proposed hypotheses—including hemodilution, increased von Willebrand factor, insufficient thrombopoietin response, decreased ADAMTS13 activity, or genetic factors—fully elucidate the condition (1, 2).

Generally, GT typically necessitates only regular platelet monitoring and clinical observation, with special therapeutic interventions being largely unnecessary during pregnancy (1). Cesarean delivery is not recommended solely for isolated thrombocytopenia in the absence of obstetric complications (2, 5).

Thrombocytopenia in traditional Chinese medicine (TCM) is primarily attributed to “blood heat.” Ancient Chinese texts, including *A Treatise on Blood Troubles, Jingyue Quanshu*, and *The Ming Yi Jia Zuo*, have emphasized the detrimental effects of “blood heat” on the collaterals, the depletion of “kidney essence,” and the subsequent progression to “yin deficiency leading to fire hyperactivity.” This condition can result in “fire consuming qi and yang,” which ultimately leads to “spleen-kidney yang deficiency” and a “qi failing to hold blood pattern.” These discussions form the foundation of TCM's understanding of hemorrhagic disorders. Contemporary clinical studies in TCM also underscore that blood heat is the predominant cause during the acute phase of thrombocytopenia (6). The conventional treatment approach employs “Rhinoceros Horn and Rehmannia Decoction” (RHRD) to “clear heat and cool blood” while stopping bleeding (7). During pregnancy, a woman's blood and “kidney essence” continuously nourish the fetus, which may lead to insufficient kidney essence in the mother and increased fetal heat (8). However, due to the strong cold properties of RHRD, its direct application in pregnancy is considered unsafe and may elevate the risk of miscarriage (7). Therefore, modifications to the classic RHRD formula are essential to accommodate the physiological characteristics of pregnancy.

We present a case of recurrent GT effectively treated with adapted Chinese herbal medicine (CHM). The individual encountered GT in two successive pregnancies, with non-pharmacological interventions proving ineffective in halting a gradual decrease in platelet levels. Following CHM therapy, platelet count normalized within the typical range for pregnancy, leading to positive outcomes for both the mother and newborn in both pregnancies.

## Case presentation

### Patient information / History

A 30-year-old woman was incidentally found to have mild thrombocytopenia during routine antenatal examination. This was her third pregnancy (G3P1A1L1). Her medical history included hypothyroidism, gallstones with cholecystitis, and prior GT. She had no history of recent viral infection, allergy, or autoimmune disease. Her paternal grandfather had reportedly died of a hematologic disorder at approximately 50 years of age, although the exact diagnosis was unknown.

Her first pregnancy ended in miscarriage at 12 weeks due to embryonic arrest. Platelet counts were not regularly monitored during that pregnancy, and thrombocytopenia was not documented before the loss. At approximately 5 weeks' gestation, she received a measles vaccine during a campus-wide immunization program organized by the university health service in response to a reported measles case. She was unaware of the pregnancy at that time.

During her second pregnancy, thrombocytopenia was first noted at 19 weeks' gestation. She was taking only levothyroxine sodium for hypothyroidism throughout her pregnancies. She was a lifelong non-smoker and consumed alcohol only occasionally. Her blood pressure and blood glucose remained within the normal range, and she had no edema or joint pain. Her diet was balanced.

### Physical examination

At 40 weeks' gestation, when she was admitted for delivery in her third pregnancy, she was well and in no acute distress. Vital signs were stable (temperature 36.6 C, pulse 88 beats/min, blood pressure 102/72 mmHg, respiratory rate 20 breaths/min). Fundal height was 36 cm and abdominal circumference 111 cm, consistent with term gestation. Fetal heart rate was 145 beats/min with a reassuring pattern. There was no pallor, jaundice, lymphadenopathy, or peripheral edema. The skin and mucous membranes showed no petechiae, purpura, ecchymoses, gingival bleeding, epistaxis, or vaginal bleeding. The liver and spleen were not palpable, and cardiopulmonary and neurologic examinations were unremarkable. Physical examination at the time thrombocytopenia was detected in the second pregnancy was likewise unremarkable, with stable vital signs and no organomegaly or bleeding manifestations.

### Examination and laboratory testing

Changes in platelet counts during the two pregnancies are summarized in Tables 1, 2. At 4 weeks' gestation, hematologic testing showed a platelet count of 116,000/μL, with an earlier onset than in her second pregnancy. Red blood cell count, white blood cell count, hemoglobin, and hematocrit were within gestational reference ranges. By 30 weeks' gestation, the platelet count had decreased to 64,000/μL.

**Table 1:** Onset and clinical course of gestational thrombocytopenia across two pregnancies.

Pregnancy	Onset of thrombocytopenia		Onset of nadir		Documented normal platelet count	
	Platelet count (×10^3^/μL)	Gestation (weeks)	Platelet count (×10^3^/μL)	Gestation (weeks)	Platelet count (×10^3^/μL)	Post-delivery (days)
2nd	132	19	72	35	158	81
3rd	116	4	64	30	190	21

In the third pregnancy, the onset of thrombocytopenia occurred at an earlier gestational stage, with a lower platelet nadir compared to the previous pregnancy.

**Table 2:** Changes in platelet counts and timing of traditional Chinese medicine administration during pregnancies.

Pregnancy	28w	29w	30w	31w	32w	33w	34w	35w	36w	37w	38w	39w	40w
2nd	–	137	–	117	–	88	80^*^	72^*^	135	125	127	110	111
3rd	73	66^*^	64^*^	84^*^	–^*^	92^*^	–	89	96	104	107	84^*^	111

w, weeks of gestation; –, data not available;

^*^indicates the weeks during which traditional Chinese medicine was administered. In the third pregnancy, TCM was given from 29 to 33 weeks and again at 39 weeks' gestation.

An extensive evaluation was conducted to determine the cause of the thrombocytopenia, excluding bone marrow examination. Blood typing showed group A, Rh-positive. Coagulation studies, including fibrinogen, prothrombin time, and activated partial thromboplastin time, were within normal limits. Thyroid function tests showed low FT3 (2.11 pg/mL; reference 2.3–4.2) and FT4 (0.78 ng/dL; reference 0.89–1.80) with elevated TSH (5.87 μIU/mL; reference 0.55–4.78); the levothyroxine sodium dose was increased to 175 μg daily. Biochemical testing demonstrated normal renal, hepatic, and cardiac function. Total cholesterol and triglyceride levels were 6.3 mmol/L (reference < 5.18) and 2.84 mmol/L (reference < 1.7), respectively, and no lipid-lowering therapy was initiated. Reticulocyte count, lupus anticoagulant, platelet aggregation, and immunoglobulin levels were within normal limits. Autoantibody screening (including anticardiolipin, antinuclear, anti-β2-glycoprotein I, anti-erythrocyte, and antiphosphatidylserine antibodies) and tests for hepatitis viruses, human immunodeficiency virus, and syphilis were all negative. Abdominal ultrasonography showed normal liver and spleen size.

### Diagnostic assessment

We first confirmed that thrombocytopenia was limited to platelets, while other blood counts, organ function tests, coagulation parameters, and abdominal ultrasonography findings remained normal throughout all three pregnancies, and no hypertension or abnormal physical signs were observed. Because platelet counts fell below 100,000/μL in the second and third pregnancies, we then evaluated causes other than GT according to current recommendations. Immune, hypertensive (preeclampsia/HELLP), thrombotic microangiopathic, drug-induced, infectious, and inherited etiologies were considered. These causes were judged unlikely because thrombocytopenia was confined to pregnancy, nadir counts remained above 50,000/μL, there was no bleeding history, blood pressure and organ function were normal, no additional cytopenias or coagulation abnormalities were present, levothyroxine was the only long-term medication, infectious and autoimmune screens were negative, and platelet counts were normal outside pregnancy and in both offspring. Taken together, this pattern of isolated moderate thrombocytopenia confined to pregnancy, with spontaneous postpartum recovery and no evidence of maternal or neonatal systemic disease, supports a diagnosis of recurrent GT with a favorable prognosis.

### Therapeutic course

GT is a benign, self-limiting condition that typically does not require specific treatment. In this case, although the patient developed recurrent moderate thrombocytopenia (nadir platelet count 64,000/μL), she had no bleeding symptoms, and therefore was managed with close observation alone. After detailed counseling, the patient elected to receive TCM as adjunctive therapy under the guidance of Professor Qingquan Liu, with the aim of stabilizing platelet counts and supporting the pregnancy, without replacing standard obstetric monitoring and management.

The TCM prescription consisted of one daily decoction containing dried *Radix Rehmanniae* (30 g), *Raw Gypsum* (45 g), *Rhizoma Anemarrhenae* (10 g), *American Ginseng* (15 g), *Honey-Fried Licorice* (10 g), *Radix Scrophulariae* (30 g), *Folium Perillae* (10 g), *Rhizoma Dioscoreae* (60 g), *Radix Paeoniae Rubra* (30 g), and *Rhizoma Polygonati* (30 g). Each daily dose was weighed and dispensed by the pharmacy of Yanqing Hospital of Beijing Chinese Medicine Hospital according to a standardized prescription. At home, the patient prepared the decoction following written instructions: the herbs were soaked in cold water for 30 min, then decocted twice, approximately 30 min each time. The two extracts were combined to yield about 600–700 mL of decoction, which was divided into three portions and taken three times daily after meals.

In the second pregnancy, once thrombocytopenia was detected, the obstetrician recommended supportive measures, including dietary therapies (peanut peel water and Wu-hong decoction) and a Chinese patent medicine (Angelica Blood-Supplementing Oral Liquid), taken intermittently from 29 to 33 weeks of gestation. Despite these interventions, the platelet count continued to fall and reached 80,000/μL at 34 weeks (Table 2). There was no abrupt change temporally related to the initiation or discontinuation of these products, making a causal role unlikely.

Although the platelet count remained within an observable range, its progressive decline near term caused marked patient anxiety regarding possible transfusion, cesarean delivery, and other complications. After shared decision-making, a daily decoction was prescribed for 13 consecutive days. The platelet count dropped transiently to 72,000/μL at 35 weeks, then increased to 135,000/μL at 36 weeks and subsequently stabilized between 110,000/μL and 135,000/μL. Immediately before delivery at 40 weeks' gestation, the platelet count was 111,000/μL. On June 13, 2020 (40 + 5 weeks of gestation), the patient delivered a healthy female infant weighing 3,100 g.

In the third pregnancy, GT recurred with earlier onset and greater severity. The platelet count had fallen to 116,000/μL by 4 weeks' gestation, and the nadir of 64,000/μL occurred at 30 weeks, lower than in the previous pregnancy (Tables 1, 2). Unlike in the second pregnancy, the patient did not receive dietary therapies or Chinese patent medicines and was treated directly with the same standardized TCM decoction once GT was identified.

Because of the earlier onset and deeper nadir, TCM therapy was initiated at 29 weeks' gestation. The decoction was taken once daily from 29 to 33 weeks and again for approximately 1 week at 39 weeks, for a total of 35 doses. Treatment was not fully continuous, with an aggregate interruption of about 7 days due to delays in follow-up and prescription refills. During this period, the platelet count increased to 107,000/μL in late gestation and was 111,000/μL at 40 weeks' gestation, immediately before delivery (Table 2). On October 18, 2022 (40 + 1 weeks' gestation), the patient delivered a healthy male infant weighing 3,600 g.

### Follow-up and outcome

Although reassured by her physicians that GT usually resolves without complication, the patient experienced considerable anxiety about miscarriage, bleeding, and neonatal hemorrhage as her platelet count declined. As platelet counts stabilized during CHM treatment, she reported reduced anxiety and greater reassurance; however, in this single case we cannot determine whether this change was attributable to CHM, hematologic improvement, or non-specific supportive care. Labor occurred spontaneously, and maternal platelet counts normalized postpartum in both pregnancies. The male neonate developed a transient cephalohematoma, which resolved without intervention. The patient adhered well to the prescribed CHM regimen and reported no adverse or unexpected events related to treatment during pregnancy, delivery, or postpartum follow-up. No additional platelet-directed therapies were given during CHM treatment in either pregnancy. As of December 2025, the older child (5 years 6 months) and the younger child (3 years 2 months) both show normal growth and neurodevelopment, with no thrombocytopenia or coagulation abnormalities.

## Discussion

This case report details a pregnant woman who experienced recurrent GT. The onset of thrombocytopenia occurred early in the first to second trimester, which is atypical for GT, as the condition typically develops in the third trimester (3, 4). The decline in platelet count was more rapid and severe than in her previous pregnancy. After treatment with TCM, the platelet count increased during the late third trimester and normalized prior to delivery. A spontaneous vaginal birth ensued without maternal or neonatal complications. Both infants exhibited normal hematological and developmental outcomes, indicating that the modified CHM regimen was safe and potentially effective in this context.

GT remains a diagnosis of exclusion, confirmed only when platelet levels normalize postpartum (2). Severe thrombocytopenia, defined as a platelet count of less than 70,000/μL, is infrequently associated with GT and typically necessitates evaluation for alternative causes, such as immune thrombocytopenia or preeclampsia (3). Women with a prior history of GT have a significantly elevated risk of recurrence in subsequent pregnancies, with a 14.2-fold increase compared to those without such a history (9). In this case, the patient's previous episode of GT, the normalization of platelet counts following delivery, and the absence of systemic disease collectively supported the diagnosis of recurrent GT.

In both pregnancies, thrombocytopenia manifested earlier than typically anticipated and progressively deteriorated as gestation progressed. Conventional non-pharmacological interventions proved ineffective; however, the administration of a modified CHM formula stabilized platelet counts and prevented further decline. Following 13 doses of the decoction during the second pregnancy, platelet levels improved from 72,000/μL to 135,000/μL. In the third pregnancy, 35 doses elevated the count from 64,000/μL to 111,000/μL prior to delivery. This favorable response indicates a potential therapeutic benefit of CHM as an adjunctive strategy in the management of GT.

From the TCM perspective, GT is associated with the manifestation of “fetal heat” due to qi deficiency and yin consumption in the latter stages of pregnancy. Therefore, the therapeutic approach should emphasize heat clearance, qi replenishment, yin nourishment, and miscarriage prevention. The modified prescription comprises *Radix Rehmanniae, Raw Gypsum, Rhizoma Anemarrhenae, American Ginseng, Radix Scrophulariae, Radix Paeoniae Rubra, Rhizoma Dioscoreae, Rhizoma Polygonati*, and *Folium Perillae*, which are believed in TCM to possess properties that clear heat, fortify qi, nourish yin, and safeguard pregnancy. From a modern pharmacological perspective, several constituents of this formula exhibit immune-modulating and haematopoietic effects that may support platelet recovery. Polysaccharides derived from Rhizoma Anemarrhenae have been shown to enhance macrophage phagocytosis and cytokine production via activation of the NF-κB and MAPK signaling pathways (10). Ginsenosides in American ginseng modulate both innate and adaptive immune responses and may help normalize dysregulated immunity (11). Rehmanniae Radix and its processed products ameliorate chemotherapy-induced blood deficiency in animal models and increase peripheral white blood cell, red blood cell and platelet counts, effects that are associated with changes in polysaccharides and non-polysaccharide constituents such as 5-hydroxymethylfurfural (12, 13), providing a plausible mechanistic basis for the observed clinical benefit.

Traditional prescribing norms and safety in pregnancy-related disorders must also be considered. Classical TCM obstetric literature consistently emphasizes “treating the mother without harming the fetus” and “prioritizing fetal protection” (8). Historical Chinese sources describe several fetus-preserving formulas, such as Baoyin Jian, Bushen Antai Yin, and Guizhi Fuling Wan, which commonly include Radix Rehmanniae, Rhizoma Dioscoreae, Colla Corii Asini, Folium Artemisiae Argyi, Radix Paeoniae Rubra, and Cortex Moutan as core components (8). In the present case, these therapeutic principles align with those of the classical fetus-preserving formulas, and these herbs have been used long-term for conditions such as hyperemesis gravidarum, threatened miscarriage, and restless fetus, with an overall acceptable safety profile in TCM clinical practice.

Current guidelines suggest that vaginal delivery is generally safe for patients with GT, regardless of platelet levels (3). However, many women still experience considerable anxiety and choose cesarean section due to concerns about thrombocytopenia. Researchers stress that cesarean delivery should be performed only for obstetric indications, rather than solely based on platelet count (14). In this instance, the application of CHM was associated with stabilization of platelet counts and a reduction in the patient's self-reported anxiety, which coincided with a safe spontaneous delivery; however, a causal relationship between CHM, psychological stress, and platelet improvement cannot be established in this single case.

To our knowledge, this is the first documented case illustrating the successful application of CHM for recurrent GT. The observed benefits indicate that CHM may function as an effective and safe adjunctive therapy in select cases, complementing standard monitoring strategies. However, this report has several important limitations. First, platelet counts during the patient's first pregnancy were not systematically monitored. Although no thrombocytopenia was documented before the miscarriage at 12 weeks and GT typically manifests in the second or third trimester, we cannot definitively determine whether GT was present in that pregnancy or whether the early pregnancy loss was related to GT, to the measles vaccination received at approximately 5 weeks' gestation as part of a campus-wide immunization program, or to other obstetric causes; any hypothesized link therefore remains speculative. Second, as a single, uncontrolled case report, the level of evidence is inherently low, there is no control group for comparison, and a causal relationship between the modified CHM regimen and platelet recovery cannot be firmly established, particularly given the sequential use of other non-pharmacological measures earlier in the pregnancies. So, well-designed prospective clinical and mechanistic studies with larger sample sizes are essential to validate the efficacy and safety of CHM for GT in both mothers and infants.

## Patient's perspective

As both the patient and the author of this report, I experienced recurrent declines in platelet count during two pregnancies, which caused considerable self-reported anxiety and fear of miscarriage or bleeding. Although I was aware that GT often resolves spontaneously after delivery, the uncertainty was emotionally challenging. When my platelet count reached its nadir, I decided to try the herbal decoction prescribed by my attending TCM physician. As I continued the decoction, I observed a gradual increase in my platelet levels, which eased my anxiety and allowed me to safely continue both pregnancies and deliver vaginally. Looking back, my dual role as a physician and as the subject of this report has deepened my appreciation of TCM as an integrative and supportive option in obstetric care. Today, my two children are healthy, and this experience has strengthened my confidence in combining modern medicine with traditional Chinese approaches to support other women facing similar challenges.

## Data Availability

The original contributions presented in the study are included in the article/supplementary material, further inquiries can be directed to the corresponding authors.

## References

[1] Park YH. Diagnosis and management of thrombocytopenia in pregnancy. Blood Res. (2022) 57:S79–85. doi: 10.5045/br.2022.2022068 PMC9057658 35483931

[2] Fogerty AE Kuter DJ. How I treat thrombocytopenia in pregnancy. Blood. (2024) 143:747–56. doi: 10.1182/blood.2023020726 37992219

[3] Habas E Rayani A Alfitori G Ahmed GE Eizouki A-NY. Gestational thrombocytopenia: a review on recent updates. Cureus. (2022) 14:e23204 doi: 10.7759/cureus.23204 35444886 PMC9010930

[4] Fogerty AE Dzik W. Gestational thrombocytopenia: a case–control study of over 3,500 pregnancies. Br J Haematol. (2021) 194:433–8. doi: 10.1111/bjh.17611 34105146

[5] ACOG. Practice bulletin no. 207: thrombocytopenia in pregnancy. Obstet Gynecol. (2019) 133:e181–93. doi: 10.1097/AOG.0000000000003100 30801473

[6] Xu H Bao J Zhu W Liu Y Wang L Zhang X. Clinical literature study of traditional Chinese medicine for primary immune thrombocytopenia. J Tradit Chin Med Lit. (2023) 41:40–3.

[7] Zhong G Yang B. Chinese Materia Medica. Beijing: China Press of Chinese Medicine (2009).

[8] Feng X Zhang T. Traditional Chinese Gynecology. Beijing: China Press of Chinese Medicine (2011).

[9] Reese JA Peck JD Deschamps DR McIntosh JJ Knudtson EJ Terrell DR . Platelet counts during pregnancy. N Engl J Med. (2018) 379:32–43. doi: 10.1056/NEJMoa1802897 29972751 PMC6049077

[10] Zhang S Zhang Q Li C Xing N Zhou P Jiao Y. A zinc-modified Anemarrhena asphodeloides polysaccharide complex enhances immune activity via the NF-κB and MAPK signaling pathways. Int J Biol Macromol. (2023) 249:126017. doi: 10.1016/j.ijbiomac.2023.126017 37517752

[11] Ratan ZA Youn SH Kwak Y-S Han C-K Haidere MF Kim JK . Adaptogenic effects of Panax ginseng on modulation of immune functions. J Ginseng Res. (2021) 45:32–40. doi: 10.1016/j.jgr.2020.09.004 33437154 PMC7790873

[12] Wang Y-Y Zhou N Shan Z-F Ke Y-Y Liu Z Liu Z-H . Metabolomic strategies and biochemical analysis of the effect of processed Rehmanniae radix extract on a blood-deficient rat model. BMC Complement Med Ther. (2022) 22:89. doi: 10.1186/s12906-022-03560-x 35337319 PMC8957163

[13] Zhang W Cui N Su F Wang Y Yang B Sun Y . Comprehensive metabolomics and network pharmacology to explore the mechanism of 5-hydroxymethyl furfural in the treatment of blood deficiency syndrome. Front Pharmacol. (2022) 12:811331. doi: 10.3389/fphar.2021.811331 35310893 PMC8931835

[14] Chen I Opiyo N Tavender E Mortazhejri S Rader T Petkovic J . Non-clinical interventions for reducing unnecessary caesarean section. Cochrane Database Syst Rev. (2018) 9:CD005528. doi: 10.1002/14651858.CD005528.pub3 30264405 PMC6513634

